# Metabolic analysis of the response of *Pseudomonas putida* DOT-T1E strains to toluene using Fourier transform infrared spectroscopy and gas chromatography mass spectrometry

**DOI:** 10.1007/s11306-016-1054-1

**Published:** 2016-06-21

**Authors:** Ali Sayqal, Yun Xu, Drupad K. Trivedi, Najla AlMasoud, David I. Ellis, Howbeer Muhamadali, Nicholas J. W. Rattray, Carole Webb, Royston Goodacre

**Affiliations:** School of Chemistry, Manchester Institute of Biotechnology, The University of Manchester, Manchester, M1 7DN UK

**Keywords:** Metabolomics, Efflux pumps, *P. putida* DOT-T1E, Toluene, Tolerance, Ornithine, FT-IR, GC–MS

## Abstract

**Introduction:**

An exceptionally interesting stress response of *Pseudomonas putida* strains to toxic substances is the induction of efflux pumps that remove toxic chemical substances from the bacterial cell out to the external environment. To exploit these microorganisms to their full potential a deeper understanding of the interactions between the bacteria and organic solvents is required. Thus, this study focuses on investigation of metabolic changes in *P. putida* upon exposure to toluene.

**Objective:**

Investigate observable metabolic alterations during interactions of three strains of *P. putida* (DOT-T1E, and its mutants DOT-T1E-PS28 and DOT-T1E-18) with the aromatic hydrocarbon toluene.

**Methods:**

The growth profiles were measured by taking optical density (OD) measurement at 660 nm (OD_660_) at various time points during incubation. For fingerprinting analysis, Fourier-transform infrared (FT-IR) spectroscopy was used to investigate any phenotypic changes resulting from exposure to toluene. Metabolic profiling analysis was performed using gas chromatography-mass spectrometry (GC–MS). Principal component—discriminant function analysis (PC-DFA) was applied to the FT-IR data while multiblock principal component analysis (MB-PCA) and *N*-way analysis of variance (*N*-way ANOVA) were applied to the GC–MS data.

**Results:**

The growth profiles demonstrated the effect of toluene on bacterial cultures and the results suggest that the mutant *P. putida* DOT-T1E−18 was more sensitive (significantly affected) to toluene compared to the other two strains. PC-DFA on FT-IR data demonstrated the differentiation between different conditions of toluene on bacterial cells, which indicated phenotypic changes associated with the presence of the solvent within the cell. Fifteen metabolites associated with this phenotypic change, in *P. putida* due to exposure to solvent, were from central metabolic pathways. Investigation of MB-PCA loading plots and *N*-way ANOVA for condition | strain × time blocking (dosage of toluene) suggested ornithine as the most significant compound that increased upon solvent exposure.

**Conclusion:**

The combination of metabolic fingerprinting and profiling with suitable multivariate analysis revealed some interesting leads for understanding the mechanism of *Pseudomonas* strains response to organic solvent exposure.

**Electronic supplementary material:**

The online version of this article (doi:10.1007/s11306-016-1054-1) contains supplementary material, which is available to authorized users.

## Introduction

Bacteria can adapt to overcome the activity of toxic substances via the application of several resistant mechanisms. An exceptionally interesting stress response of *Pseudomonas putida* strains to toxic substances is the induction of efflux pumps, which, as their name suggests, remove toxic substances from the bacterial cell out to the external environment (Fernandes et al. 2003; Poole 2007; Ramos et al. 1998). This mechanism is probably the most important process that plays an absolutely crucial role in bacterial adaptation mechanisms. The development of solvent–tolerant microorganisms that are able to grow in the presence of toxic organic solvents are useful in many applications, for example in environmental bioremediation (Nicolaou et al. 2010) and biocatalysis where organic solvents are often used to dissolve the substrate and product (Ellis and Goodacre 2012).

Bioremediation is an incredibly important form of waste management that involves the conversion of harmful substances into non-harmful end products via the use of microorganisms (Bustard et al. 2000, 2002; Gupta et al. 2006; Pandey et al. 2009; Zhao and Poh 2008). Solvent tolerance is an adaptive process, as it is possible to make the bacteria tolerant to harsh environments through a number of reported methods. One approach to adapt the characteristics of microbial cells to unfavourable culture conditions has included the pre-exposure of bacterial cultures to low concentrations of toxic solvent (Ramos et al. 1998; Xin et al. 2009). Alternative methods such as genetic engineering can also be used to produce altered strains with superior tolerance characteristics, and this can be achieved through transformation of the microorganism to include a plasmid that confers degradation properties (encodes key enzymes) to specific toxic solvents (Horikoshi et al. 2011). This would allow for increased decontamination rates, so an understanding of the mechanisms of solvent toxicity is of great importance in order to explore microorganisms that exhibit sufficient tolerance, thereby enabling them to serve as bioremediation agents for specific chemical contaminants.

Whole-cell biocatalysis in two-phase systems containing an organic phase is an application for the production of specialty or fine chemicals (Heipieper et al. 2007; Neumann et al. 2006; Sardessai and Bhosle 2004). In many instances, the initial material and/or the end-product can display some toxicity to the biocatalyst, which of course leads to limited production yields or affect the overall performance (which could be biotransformation specificity) of biocatalysis. Thus, the ability to exploit these microorganisms to their full potential requires a deeper understanding of the interactions between the bacteria and organic solvents, which is an important research goal. Changes identified in the microbial metabolome can be considered to be hypothesis generating and as such can inform our biochemical knowledge (Goodacre et al. 2004; Kell and Oliver 2004). Observed metabolite changes can prove to be indicative of novel adaptation mechanisms, or may support postulated adaptation mechanisms for which there are little evidence to date.

In this study, the effect of the sudden addition of toluene to *P. putida* DOT-T1E, and two mutants of this strain—*P. putida* DOT-T1E-PS28 (lacking the TtgGHI pump) and *P. putida* DOT-T1E-18 (lacking the TtgABC pump)—grown in LB medium, in the presence/absence of toluene via gas phase has been investigated. Metabolomics strategies were applied, specifically metabolic fingerprinting (Ellis et al. 2007) employing FT-IR spectroscopy (Ellis and Goodacre 2006) in order to identify general phenotypic alterations in bacterial cultures exposed to toluene, and metabolic profiling using GC–MS to investigate any metabolome changes in response to solvent stress. The data sets generated via these approaches were explored further using multivariate analysis methods in order to model the metabolic effect of organic solvents on microbial species.

## Materials and methods

### Bacterial strains and culture medium

Three strains of *P. putida* were chosen for this study to investigate the response of bacteria to toluene stress and these are listed in Table 1, and were sourced from the Juan Luis Ramos lab (Consejo Superior de Investigaciones Cientificas, Estacion Experimental del Zaidin, Department of Biochemistry and Molecular and Cellular Biology of Plants, Granada, Spain, http://www.eez.csic.es/?q=en/node/51). Nutrient agar (NA) and lysogeny broth (LB) were used for cultivation of bacteria. NA was prepared as follows: peptone 5 g/L, beef extract 3 g/L, sodium chloride 8 g/L, 12 g/L of agar no. 2. LB medium contained: tryptone 10 g/L (Formedia, Hunstanton,UK), yeast extract 5 g/L (USP, Cleveland, USA) and NaCl 10 g/L.

**Table 1:** *P. putida* strains used in this study

*P. putida* strains	Relevant characteristics^a^	References
DOT-T1E	Ap^r^ Rif^r^ Tol^r^	Ramos et al. (1995)
DOT-T1E-PS28	Rif^r^ Sm^r^ *ttgH*::VSm	Rojas et al. (2001)
DOT-T1E-18	Rif^r^ Km^r^ *ttgB*::’*phoA*-Km	Ramos et al. (1998)

^a^ Resistance to *Ap* ^*r*^ ampicillin, *Rif* ^*r*^ rifampin, *Sm* ^*r*^ streptomycin, *Km* ^*r*^ kanamycin, *Tol* ^*r*^ toluene; note that the mutants have different deletions in the two genes involved in toluene tolerance (*ttg*)

### Cultivation of bacteria and culture conditions

All three strains of *P. putida* DOT-T1E were sub-cultured in triplicate on agar plates in order to obtain pure single colonies. Cells were grown in LB and the axenic cultures were incubated overnight with horizontal shaking in an orbital incubator (Infors HT Ltd, UK) at 30 °C and 200 rpm.

### Growth in response to toluene, sample collection and analysis

*P. putida* cells were normalised to an optical density (OD) of 0.1 and then incubated in an orbital shaker for 1 h at 30 °C and 200 rpm. At this point, cultures were divided into two groups: one was kept as a control and for the other toluene was supplied via gas phase for only 30 min in which an evaporation tube containing 100 µL of toluene was used in order to avoid direct contact with the culture. The culture flasks containing 50 mL of LB medium were sealed with Suba-Seal to prevent toluene leakage and then incubated for an additional 4 h. The concentration of toluene in the flask was approximately 12.5 mM (0.125 % (v/v)) under these culture conditions. Once cell cultures reached the mid-log phase, the cultures were split into two halves; to one 0.1 % (v/v) toluene was added and the other was kept as a control, and then cell cultures were incubated for additional 7 h. The tested concentration of toluene is below the minimum inhibitory concentrations (MICs) which are 5, 0.8 and 0.7 % (v/v) for DOT-T1E, DOT-T1E-PS28 and DOT-T1E-18 respectively (Table S1).

#### Bacterial growth profiles

During the time-course of 12 h incubation, (100 µL) samples were collected at various time points (0, 1, 3, 5, 7, 9, 11 and 12 h) in triplicate from each flask and from each of the exposure conditions (i.e., positive and negative groups) for OD measurement at 660 nm (OD_660_).

#### Analysis of biomass samples by FT-IR spectroscopy

Aliquot (2 mL) samples were collected and centrifuged at 11,500×*g* for 5 min at 4 °C (ThermoFisher CR3.22, UK). The supernatant was removed, while the cell pellets were washed twice with 2 mL of sterile physiological saline solution (0.9 % NaCl) (Fisher, UK) and centrifuged again. The supernatant was discarded prior to storage of the cell pellet at −80 °C for further analysis (Muhamadali et al. 2015a). The OD_660_ of samples were recorded for normalisation and the procedure was conducted in three replicates. Samples were defrosted on wet ice and then normalised and resuspended in saline solution and vortexed briefly. Normalised samples were randomised and spotted as 20 µL aliquots in triplicate onto a 96-well silicon FT-IR plate. The silicon plates were then dried in a desiccator at 25 °C for 7 h.

The silicon plate was loaded onto a motorised microplate module HTS-XT™ under the control of a computer programmed with OPUS software version 4. Triplicate spectra were obtained from each sample, resulting in a total of nine spectra per biological sample, therefore a total of 324 spectra were collected. Spectra were acquired by employing a Bruker Equinox 55 FT-IR spectrometer (Bruker Optics, Banner Lane, Coventry, UK) as described by Winder and co-workers (Winder et al. 2006). Transmission measurements of the samples were acquired and converted to absorbance spectra, using a deuterated triglycine sulfate (DTGS) detector over the wavenumber range 4000–600 cm^−1^, with a resolution of 4 cm^−1^, 64 scans were co-added and averaged to improve the signal-to-noise ratio.

The IR data were converted to ASCII format using OPUS reader software and imported into Matlab version. 2012 (MathWorks, Natick, MA). Prior to analysis, atmospheric CO_2_ vibrations in the 2400–2275 cm^−1^ region were removed and the spectra were scaled using extended multiplicative signal correction (EMSC) (Martens et al. 2003).

Principal component analysis (PCA) was used to generate sets of latent variables (PCs) that retain the most important variance in the data whilst reducing the dimensionality (Wold et al. 1987). In addition, discriminant function analysis (DFA) was then employed, which is a supervised method that discriminates groups by a priori knowledge of sample origin. DFA attempts to maximise the differences between the known groups (classes) whilst minimising the differences within the class (Gromski et al. 2015; Johnson et al. 2003; Macfie et al. 1978). PC-DFA was conducted utilising PCs 1–10, and the class structure for the DFA algorithm was based on the biological replicates of samples from the same conditions.

#### Metabolite profiling

##### Sample collection and metabolic quenching

Samples were collected as 15 mL aliquots at several time points (0, 10 and 60 min) in the absence and presence of different toluene conditions (0 min refer to the point immediately *before* the addition of toluene shock). The metabolic activity of the collected samples were immediately quenched by adding 30 mL of cold (−50 °C) 60:40 (v/v) methanol:water followed by centrifugation at 3000×*g* for 10 min at 1 °C. After the centrifugation the supernatant was discarded, while the cell pellets were stored at −80 °C prior to metabolite extraction (Winder et al. 2008).

##### Metabolite extraction

An aliquot (750 µL) of cold (−20 °C) 80:20 (v/v) methanol:water was added to the biomass and then transferred into a 2 mL Eppendorf tube, followed by three freeze–thaw cycles to extract the intracellular polar metabolites into the polar phase. The samples were then pelleted by centrifugation (13,500×*g*, 3 min, 4 °C) and the supernatant stored on dry ice. This procedure was undertaken twice on the cell pellets and both extracts were combined and kept on dry ice.

Aliquots (1400 µL) of intracellular extracts were normalised according to OD_660_, followed by the preparation of a quality control (QC) sample (Dunn et al. 2011; Fiehn et al. 2008). The QC sample was prepared by transferring an equal volume of sample (100 µL) into a 15 mL centrifuge tube. Internal standard solution (0.2 mg mL^−1^ succinic-*d*_*4*_ acid, 0.2 mg mL^−1^ benzoic-*d*_*5*_ acid, 0.2 mg mL^−1^ lysine-*d*_*4*_, and 0.2 mg mL^−1^ glycine-*d*_*5*_) was added (100 µL) to all samples. The samples were then dried for 16 h in a speed vacuum concentrator (concentrator 5301; Eppendorf, Cambridge, UK), and stored at −80 °C prior to GC–MS analysis.

##### GC–TOF–MS analysis

Metabolite samples were removed from −80 °C storage and re-dried for 3 h in a concentrator prior to derivatisation, in order to remove any moisture absorbed by the sample during thawing, which could interfere with derivatisation process. Samples were derivatised for GC–MS following a two stage process as described previously (Wedge et al. 2011). Briefly, an aliquot (50 µL) of *O*-methylhdroxylamine hydrochloride solution (20 mg mL^−1^ in pyridine) was added to all samples. The samples were then heated using a heating block at 65 °C for 40 min followed by addition of 50 µL of MSTFA (*N*-methyl-trimethylsilyltrifluoroacetamide) and then heated for 40 min at 65 °C. An aliquot (20 µL) of retention index solution (*C*_*10*_/*C*_*12*_/*C*_*15*_/*C*_*19*_/*C*_*22*_*n*-alkanes) was added for chromatographic alignment.

The gas chromatography time-of-flight mass spectrometry (GC–TOF–MS) method was used to analyse the derivatised samples in a random order. The instrument was operated using an Agilent 6890 GC coupled to a LECO Pegasus III TOF mass spectrometer (Leco, St. Joseph, MI, USA), as described previously (Begley et al. 2009; Dunn et al. 2011) which follows metabolomic standards initiative (MSI) guidelines (Sumner et al. 2007). QC samples were employed prior to statistical analysis as described from a previous report (Wedge et al. 2011), in order to provide quality assurance of the data by the evaluation and removal of mass features that exhibit high deviation within the QC samples.

##### Data analysis

All data collected in this study were analysed on Matlab version 2014a (Mathworks, Natick, MA). The data were analysed using multi-block PCA (Smilde et al. 2003) with three different types of blockings. Strain | time × condition blocking was the first type of blocking and it partitioned the data into nine blocks, each block had the samples taken under the same toluene condition and at the time points while the strains were matched across blocks. The second type of blocking was time | strain × condition blocking. This blocking partitioned the data into 12 blocks, each block had the samples of the same strain, same condition of toluene while the time points were matched across blocks. The last type of blocking was condition | strain × time blocking which partitioned the data into six blocks (this type of blocking did not include the samples at 0 min since this time point refers to the point immediately before the addition of toluene shock), each block contained all the samples from the same strain with the same time point, while the conditions of toluene were matched across blocks. Such blocking allows for the detection of the effect of each of the factors of interest (the factor which matched across different blocks, e.g. strain | time × condition blocking was used to detect the differences between different strains) separately without the inference from others by MB-PCA (Xu and Goodacre 2012). A total number of 116 unique GC–MS peaks were detected. The natural logarithm (ln) was used on the peak area of these peaks. Data were then mean-centred, auto-scaled then subjected to MB-PCA. The most significant variables were recognised by choosing the most predominant averaged block loadings and *N*-way Analysis of Variance (*N*-way ANOVA). These results were visualised and compared using box-whisker plots.

All FT-IR and GC–MS data are freely available at MetaboLights (http://www.ebi.ac.uk/metabolights/): study identifier MTBLS319.

## Results and discussion

### The effect of toluene on the growth of *P. putida* strains

**Fig. 1:**
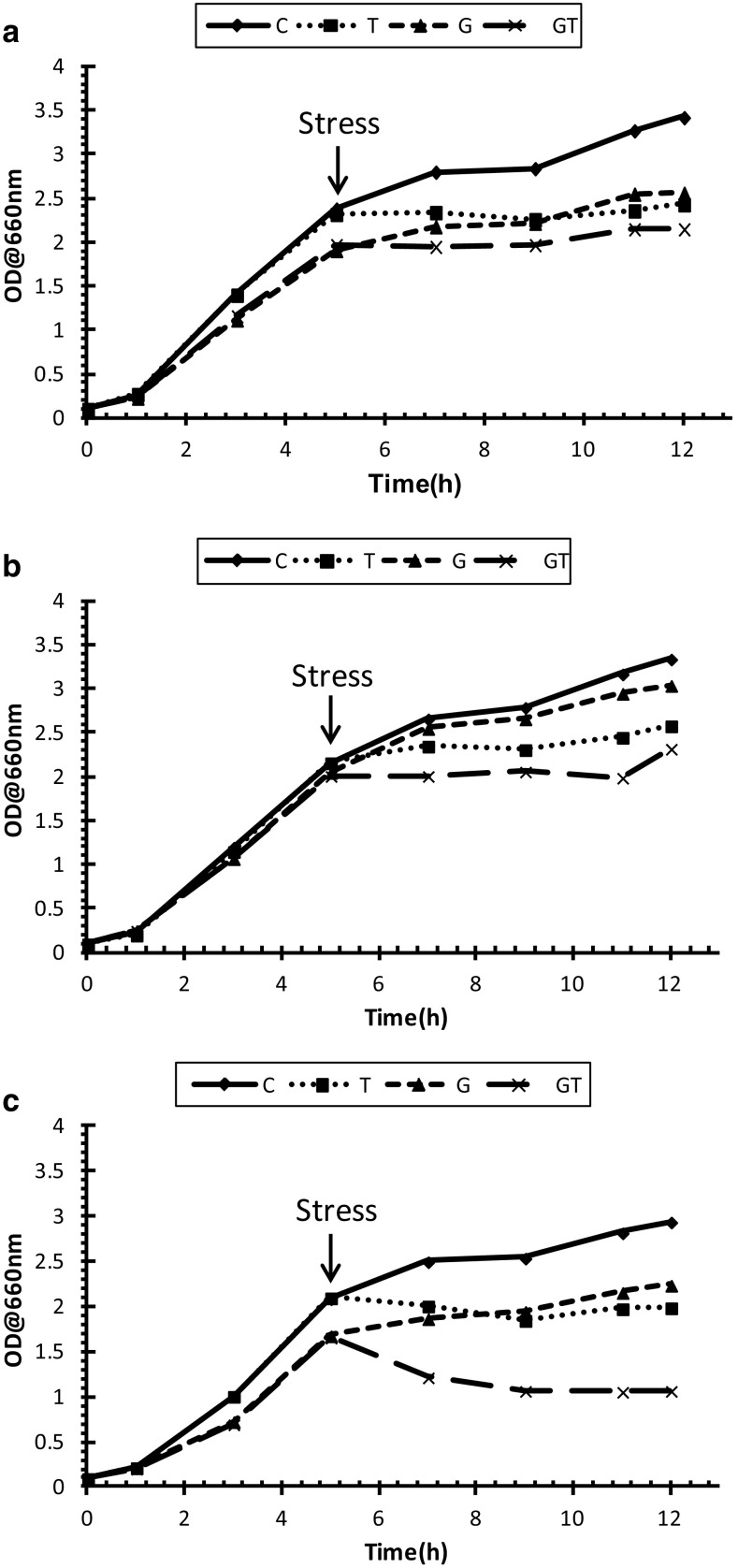
Growth curves of: **a** the wild-type DOT-T1E, **b** the mutant DOT-T1E-PS28, and **c** the mutant DOT-T1E-18 in LB medium with and without toluene. *Symbols* represent different growth conditions. Control cultures—no toluene (*diamonds*), exposed cultures to 0.1 % (v/v) toluene (*squares*), toluene gas (*triangles*), toluene gas and 0.1 % (v/v) toluene (*crosses*). A 1/10 dilution of 100 µL samples was prepared in order to determine the turbidity at 660 nm. 5 h time point is the point immediately before any toluene addition

Growth of *P. putida* cells was examined in liquid culture medium, once cells were pre-grown on LB medium with and without toluene via the gas phase. After this *P. putida* cultures were challenged with sudden shock of 0.1 % (v/v) toluene which is below the minimum inhibitory concentration (MIC) (see Table S1). Growth curves from *P. putida* cells can be seen in (Fig. 1a–c). Generally, it can be clearly noted that there is a demonstrable effect caused by the sudden addition of toluene in the flask cultures. Both non-induced and induced cells were sensitive to the sudden shock of toluene, and the final biomass of the samples decreased (as indicated by a decreased final OD reading and the final turbidity measurement being lower than negative control cells) over the 12 h incubation time-course. Our results would suggest that this decrease in the biomass could be due to energy consumption as solvent tolerance is an energy intensive process, and not due to bacterial cell death as the concentration used was below MIC. One study showed the effect of sub-lethal toluene concentrations on the growth yields of a solvent-tolerant *Pseudomonas* strain (Isken et al. 1999). It was found that cultures exhibited lower yields once grown in the presence of toluene and the biomass was decreased linearly with increasing toluene concentrations, suggesting that high levels of energy are extremely important for solvent tolerance in order to protect the cells from excessive damage.

Furthermore, the wild type *P. putida* DOT-T1E and the mutant *P. putida* DOT-T1E-PS28 were less sensitive to 0.1 % (v/v) toluene, compared to the mutant *P. putida* DOT-T1E-18, when cells were pre-grown in the absence or presence of toluene supplied via the gas phase. In addition, to assess the accumulation of toluene and the role of efflux pumps in *P. putida* DOT-T1E cells, HPLC was used to measure toluene levels in bacterial cells. Figure S1 shows the chromatograms obtained for reference toluene and bacterial cultures. To quantify the level of toluene in *P. putida* cells, a calibration curve for toluene was generated (Fig. S2). As observed in Fig. S3 and Table S2, the level of toluene in the mutant DOT-T1E-PS28 and DOT-T1E-18 were twofold and sevenfold higher compared to the wild-type DOT-T1E. Therefore, these results would clearly suggest that the TtgABC pump plays a more important role in toluene efflux than the TtgGHI pump, and these observations are in agreement with previous studies which show that the TtgABC pump is the main extrusion pump for strain tolerance as it has the ability to extrude solvents and antibiotics (Duque et al. 2007; Roca et al. 2008; Teran et al. 2003). The next stage was to assess the bacterial biochemical changes during toluene stress.

### FT-IR spectroscopy of collected biomass samples

**Fig. 2:**
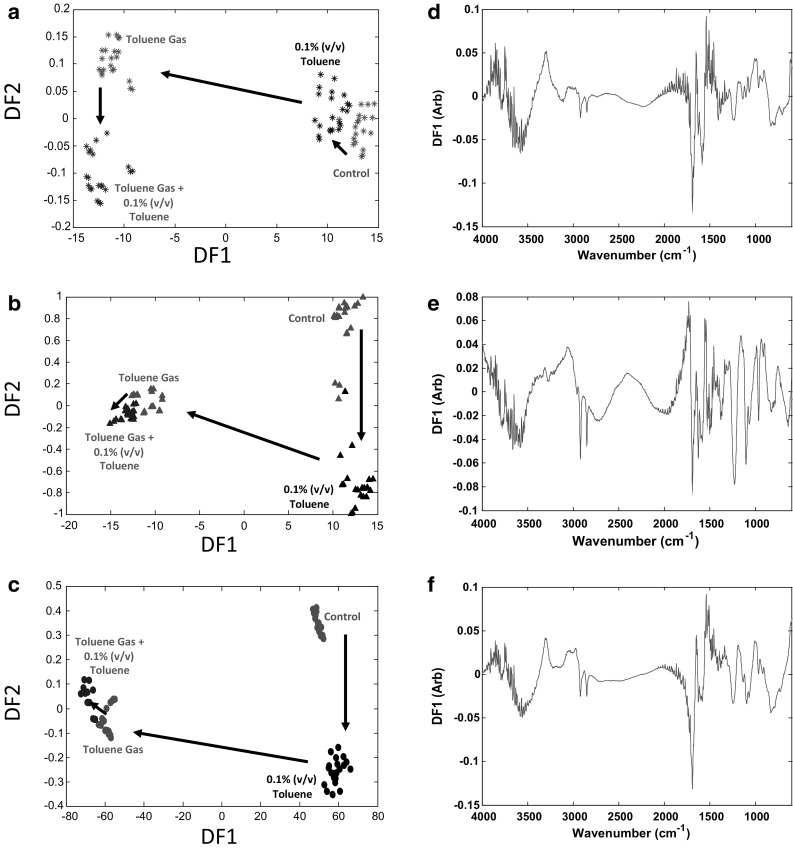
PC-DFA scores plots of FT-IR data for three different strains of *P. putida* strains upon toluene stress. Symbols show different strains. **a** *P. putida* DOT-T1E (*stars*) and the first 10 PCs with a total explained variance (TEV) of 99.94 % were used for the DFA, **b** *P. putida* DOT-T1E-PS28 (*triangles*) and PCs 1–10 with TEV of 99.93 % were used for the DFA, **c** *P. putida* DOT-T1E-18 (*circles*) and first 10 PCs with TEV of 99.90 % were used for the DFA. *Colours coding* represent different conditions: control cultures—no toluene (*red*), cultures exposed to 0.1 % (v/v) toluene (*black*), toluene gas (*brown*), toluene gas and sudden 0.1 % (v/v) toluene (*blue*). *Arrows* indicate the direction of shift because of the presence of toluene. **d** PC-DFA loadings plot for *P. putida* DOT-T1E, **e** DOT-T1E-PS28 and **f** DOT-T1E-18. Significant loadings were assigned to bacterial proteins and lipids

FT-IR was employed to assess and compare the metabolic fingerprint of *P. putida* strains under the examined conditions. All FT-IR spectral data were subjected to the supervised method of PC-DFA and the resultant DFA scores plots are displayed in Fig. 2a–c. It is evident that cells induced to toluene (vapour) cluster together significantly and separately from the non-induced cells, and also a noticeable shift was observed in the exposed cells to 0.1 % (v/v) toluene from the control cultures. This clustering pattern would suggest that toluene stress had an obvious effect on the cell cultures and may cause alterations to the phenotype of cells. In addition, it is clear to be seen that non-induced cultures collected from the 0.1 % (v/v) toluene exposed cells cluster separately from the positive cultures in the mutant strains compared to the wild-type. These clustering patterns could suggest that the parent strain was less sensitive to 0.1 % (v/v) toluene in comparison to the mutants, indicating the important role of the activity of efflux pumps in response to toluene. To ensure that the model quality is of a high standard, and that the obtained subsequent conclusions drawn from the data are valid, these PC-DFA models were validated by test set projection as can be seen in Fig. S4.

As these results show significant differences and are valid (Fig. S4), the FT-IR spectra were investigated further using the loadings plots for PC-DFA and wavenumbers with significant loadings in the PC-DFA were identified. The loadings plots of *P. putida* strains for the first discriminant functions for the three strains are shown in (Fig. 2d–f). According to these loading plots the largest variances are observed between wavenumbers 1750–1550 cm^−1^ which is attributed to changes in the protein components of the cells; most notably amide I (C=O stretching at 1690–1620 cm^−1^) and amide II (combination of C–N stretching and N–H bending at 1550 cm^−1^). However, we also see changes in the spectral regions of 2930–2850 cm^−1^ in which we would expect C–H stretching from fatty acids to occur. These results would indicate that metabolites within the amide and fatty acid regions contributed to differential responses to toluene challenge. Therefore, the most significant effects of toluene stress on bacterial cultures would be associated with changes in proteinaceous and lipid components of bacteria. Indeed, previous proteomic analysis has revealed that a number of proteins were up-regulated as a result of exposure of *P. putida* DOT-T1E or S12 strains to toluene stress (Segura et al. 2005; van der Werf et al. 2008; Wijte et al. 2011). In addition, changes in lipid compositions of DOT-T1E and S12 strains have also been shown to be involved in solvent tolerance in order to adapt membrane fluidity to the presence of toluene (Bernal et al. 2007; Ramos et al. 1997). Unsurprisingly, the interpretation of FT-IR spectra showed the most significant changes in the frequency of the proteins and lipid components. Our observations can deduce that some proteins were up-regulated and also lipid compositions were altered in response to toluene by *P. putida* DOT-T1E strains. As the FT-IR results showed that there was an effect of toluene on the phenotype of *P. putida* strains, GC–MS was employed as a metabolic profiling approach to specifically identify the significant metabolites.

### Metabolic profiling with GC–MS

The aim of metabolite profiling is to measure all, or more realistically a subset of the metabolites present in the sample, and several analytical platforms can be employed for metabolite profiling (Dunn 2008; Ellis and Goodacre 2012; Fiehn 2002). In recent years much attention has been focused on studying the stress responses in microorganisms employing metabolomics-based approaches (Allwood et al. 2015; Brito-Echeverria et al. 2011; Kol et al. 2010; Muhamadali et al. 2015a, b). The knowledge of variations within the metabolome following exposure to a stressor could lead to a more in-depth understanding of strain stress responses within these bacteria, therefore we employed GC–MS for metabolic profiling.

**Fig. 3:**
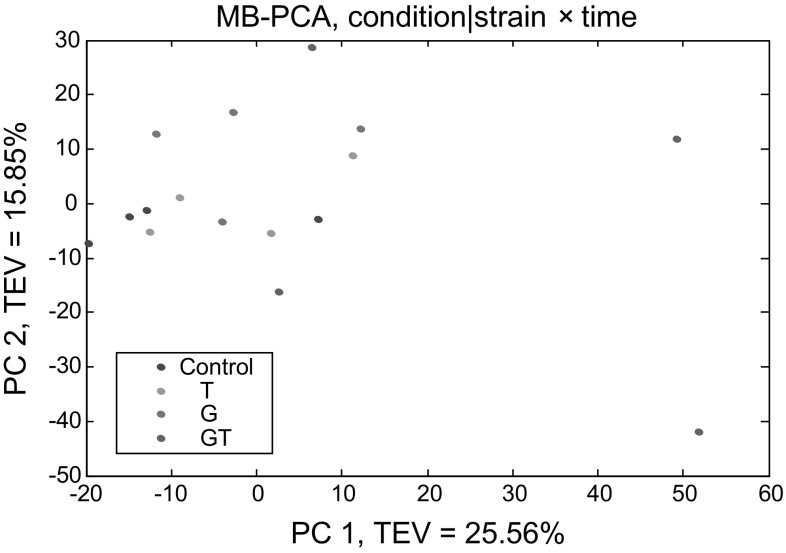
MB-PCA scores plot of GC–MS data showing the effect of different conditions of toluene on *P. putida* strains. *Colours* represent different conditions of toluene. Control with no toluene (*blue*), cells exposed to 0.1 % (v/v) toluene (T; *green*), toluene gas (G; *pink*), and toluene gas and 0.1 % (v/v) toluene (GT; *red*)

**Fig. 4:**
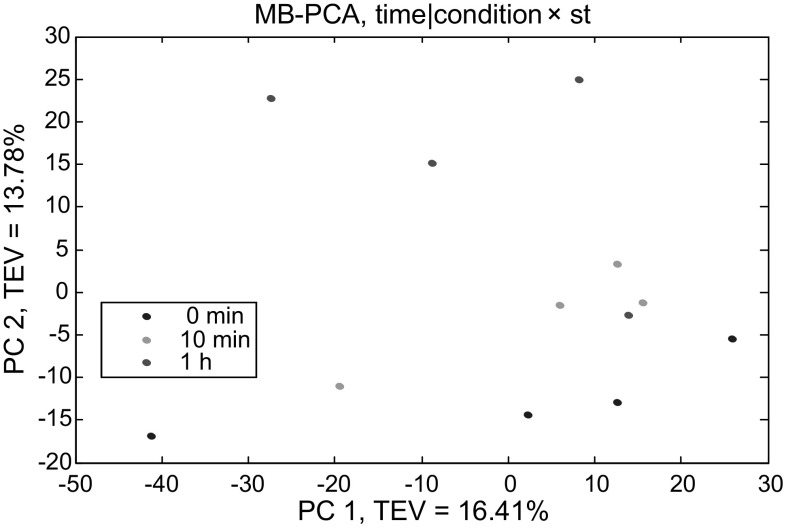
MB-PCA scores plot of GC–MS data showing the effect of different time points on *P. putida* strains. *Colours* represent different time points. 0 min is *blue* which refers to the point immediately before toluene addition, 10 min in *green* and 1 h in *red* refer to the points after 10 min and 60 min of the addition of 0.1 % (v/v) toluene

As we have multiple interacting factors (viz. strain, condition, and time) we used multi-block PCA to allow these factors to be analysed independently, an approach we have used successfully before (Xu and Goodacre 2012). Therefore to investigate the general metabolic effect of toluene on *P. putida* cells, MB-PCA with condition | strain × time and time | condition × strain were undertaken and the results are presented in Figs. 3 and 4 respectively. As can be seen in Fig. 3, a slight separation between the non-exposed and exposed cultures to toluene is observed, indicating that there are metabolic changes caused by toluene. An MB-PCA score plot was also conducted to investigate the metabolome alteration of *P. putida* cells during the time course of the exposure and as can be seen in Fig. 4, the scores of the 0 min time point are located in the bottom right and as time of incubation increases the cluster spreads from bottom to top. This clearly indicates that *P. putida* cells have different metabolic responses at different time points. Following MB-PCA, the next objective was to identify which metabolites were significantly changed between different conditions or time points.

**Fig. 5:**
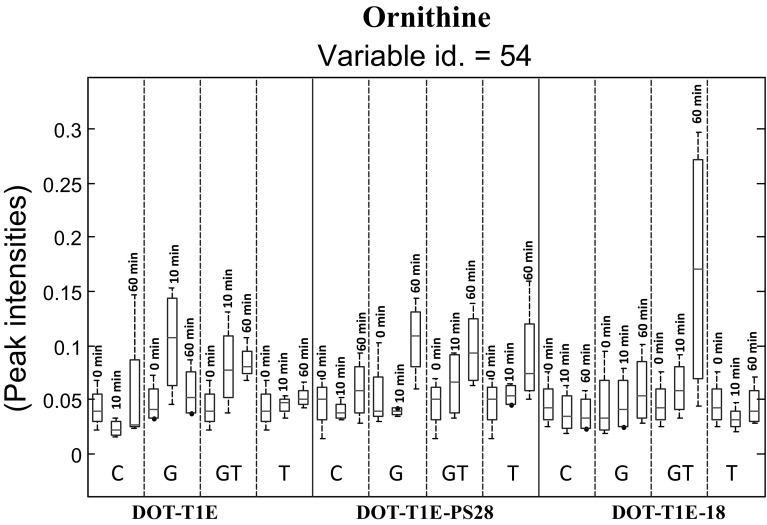
Box-whisker plot showing the alterations in ornithine levels (variable id 54) in control and cells exposed to toluene for four biological replicates. The *red lines* represent the median m/z intensity. Box plot represents the data for three *P. putida* strains, four conditions of toluene and three time points, *dashed lines* separate different conditions of toluene and *solid line* separates different strains. Code: control—no toluene (C), cells exposed to 0.1 % (v/v) toluene (T), toluene gas (G), and toluene gas and 0.1 % (v/v) toluene (GT)

The loading plots were inspected for the most significant peaks (Table S3) and *N*-way ANOVA statistical test were conducted (Table S4), and the top significant features were selected whose *p* value computed by *N*-way ANOVA was below 0.05 and also its corresponding false discovery rate (FDR) was below 0.05. A list of the identified metabolites can be seen in Table S5. Interestingly, both statistical methods for condition effect (exposure to solvent) suggested variable 54 as a highly significant feature which was identified as ornithine by our in-house library (Sumner et al. 2007; Brown et al. 2009). Figure 5 shows that the levels of ornithine in the non-exposed cultures to toluene are significantly lower than exposed cells. This might indicate a requirement for this metabolite for strain tolerance. It is noteworthy that in the induced cultures to toluene, 60 min following exposure to toluene, the level of ornithine was the highest among the other conditions and this could reveal that under this condition, the cultures exposed longer to low concentrations of toluene may allow for the cells to resist harsh conditions and sudden shock of stress. This could result in increasing the production level of ornithine in order to cope with toluene stress. In contrast, our previous study examined the effect of propranolol on *P. putida* DOT-T1E cells which showed that the ornithine was only produced following the exposure of *P. putida* strains to propranolol but was not found in the control, which also suggests that ornithine could be linked directly to the generalised strain tolerance to toxic assault (Sayqal et al. 2016). Our observations in this present study would infer that the production of ornithine in the control cultures is due to oxidative stress resulting from sealing the flask cultures with Suba-Seal rubber to prevent the toluene leakage from the flask in the exposed cultures.

Subsequently, we tested whether plugging the flask with Suba-Seal reduced the level of oxygen in the flask cultures. To ensure that the cells used for metabolic profiling analysis were exposed to oxidative stress, the flask cultures were plugged with both cotton wool and Suba-Seal rubber and then incubated at 30 °C and 200 rpm for 24 h. The resultant growth curves are shown in Fig. S5. The cultures that were plugged with Suba-Seal rubber exhibited slower growth compared to the control groups over the incubation time, which may indeed be a result of reduced oxygen level in the cultures promoting slower growth.

Mahendran and colleagues demonstrated the effect of using various oxygen regimes on growth patterns of *Pseudomonas* spp. for the biodegradation of aromatic hydrocarbons, and their results showed that all strains have the ability to grow and degrade the aromatic hydrocarbons under varying oxygen levels but in a differing manner (Mahendran et al. 2006). In the DOT-T1E strain, the presence of solvents resulted in the up-regulation of several terminal oxidase genes, suggesting adaptation by *P. putida* DOT-T1E to solvents as well as to variable aerobic and microaerobic conditions, a situation that demands the consumption of energy in order to cope with the stress (Rojo 2010). In the DOT-T1E (as well as the S12 strain they studied), proteomics analyses revealed that the up-regulation of several proteins of the TCA cycle involved in energy production upon exposure to solvents indicates a requirement for enhanced metabolism and high energy demand in order to power efflux pumps (Segura et al. 2005; Udaondo et al. 2012). As previously reported for *P. putida*, ornithine could be synthesised in several steps from glutamate through the TCA cycle where most of the energy production occurs (Antonia Molina-Henares et al. 2010). Our observation would suggest that the ornithine production in the presence of toluene is interesting, as the *P. putida* strain might activate the metabolic pathways for ornithine to demand energy to power efflux pumps due to the high activity of efflux pumps. Alternatively this may be related to other metabolic pathways that are important in response to toluene in *P. putida*.

In this work, we aimed to investigate similarities and differences in the levels of metabolites in the central metabolic pathways between the wild type and the mutants in *P. putida* DOT-T1E when cells had been pre-grown on LB medium in the absence or presence of toluene supplied via the gas phase, and these cells were then challenged with 0.1 % (v/v) toluene.

**Fig. 6:**
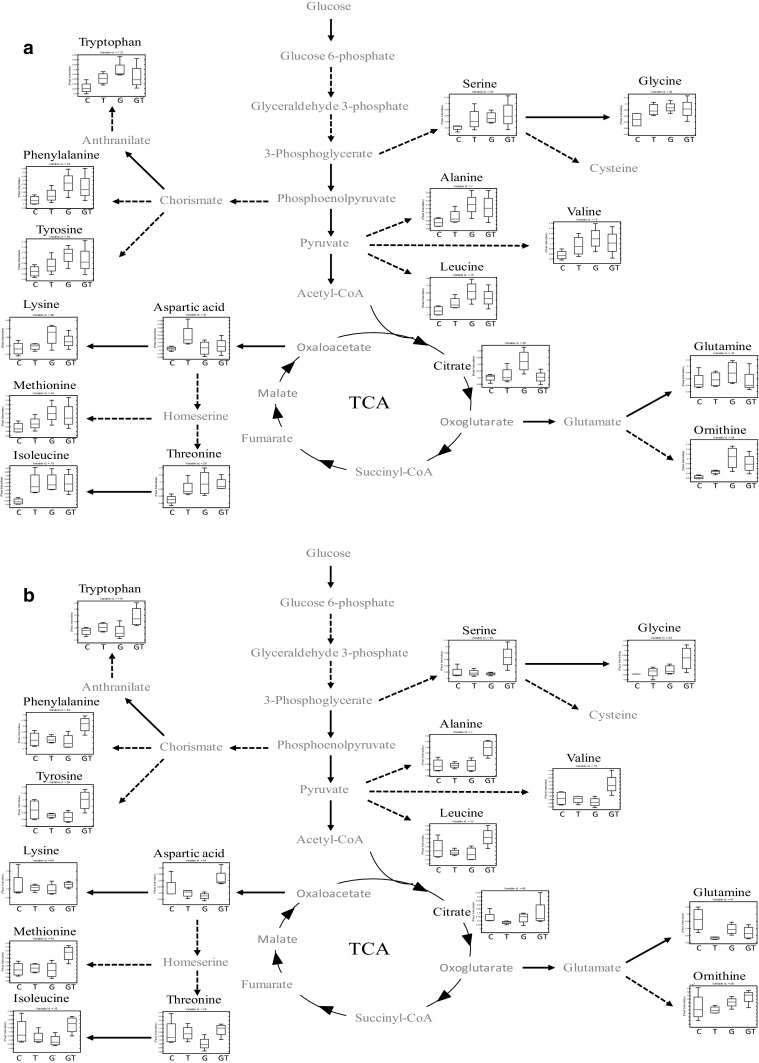
Schematic metabolic diagram of central carbon metabolism in *P. putida* DOT-T1E adapted to toluene. Metabolites were detected and identified by GC–MS. Metabolites indicated in *black* were observed, while metabolites indicated in *grey* were not detected. **a** Represent the level of metabolites at 10 min after toluene exposure, and **b** at 60 min. Box-whisker plot showing the changes in metabolite levels in control and cells exposed to toluene for four biological replicates. The *red lines* indicate the median m/z intensity. Code: control—no toluene (C), cells exposed to 0.1 % (v/v) toluene (T), toluene gas (G), and toluene gas and 0.1 % (v/v) toluene (GT)

Rather than just concentrating on a single metabolite difference we also studied the level of metabolites for each bacterial strain independently. As we used GC–MS for untargeted metabolic profiling we were able to identify the changes in the levels of metabolites during toluene stress in central carbon and nitrogen metabolism. Schematic summaries of central metabolic pathways in response to toluene in *P. putida* DOT-T1E, *P. putida* DOT-T1E-PS28, and *P. putida* DOT-T1E-18 are shown in Figs. 6a, b, S6A, B and S7A, B, respectively. In general, the mutants had similar patterns in the levels of metabolites compared to the wild type.

A direct observation from the metabolomic analysis is that the pool of amino acids (e.g. serine, glycine, alanine, valine, leucine, tryptophan, phenylalanine, tyrosine, lysine, methionine, isoleucine, threonine and ornithine) increased under the exposure of cells to toluene conditions at 10 min (shown in panels A of these figures) followed by an increase or decrease at 60 min (panel B). The levels of the most detected metabolites in the toluene adapted cells followed by toluene shock (GT) were higher than in the non-adapted cells. This observation would indicate that in the presence of vapour toluene, cells might activate metabolic pathways that are involved in toluene tolerant mechanisms prior to toluene shock. With the result of the production of higher levels of metabolites in comparison to non-induced cells, in order to cope with toluene stress and prevent cell death. A previous study found that exposure of *P. putida* DOT-T1E cultures to toluene supplied via the gas phase resulted in more rigidity of the cell membranes compared to non-exposed cultures (Ramos et al. 1998), and this may also be observed in this study from the FT-IR analyses (Fig. 2). In addition, under the same conditions it was revealed that the expression level of the *ttg*GHI operon in *P. putida* DOT-T1E was higher in the induced cells in comparison to non-induced cells (Rojas et al. 2001). Therefore, our observation would suggest that an increased pool of amino acids would illustrate the participation of metabolites in response to toluene stress. However, 60 min following the exposure to toluene, the glutamine levels were slightly deceased in exposed cells compared to the control. It is possible that the level of glutamine was decreased as glutamate might be converted into ornithine instead of glutamine, as ornithine would be the key stress-responsive metabolite involved to cope with stresses following perturbation by toluene.

## Conclusion

Our study shows that metabolic fingerprinting and profiling of *P. putida* cells by FT-IR and GC–MS analyses provides valuable information on the biological changes in these bacterial cultures upon toluene exposure. The growth profiles demonstrated the effect of toluene on bacterial cultures and the mutant *P. putida* DOT-T1E-18 was more sensitive to toluene compared to the other strains. This indicates that efflux pumps play a crucial role in strain tolerance, as also illustrated by the LC analyses of the toluene accumulation in the bacterial cells of three strains when exposed to toluene. The data collected by FT-IR shows that PC-DFA scores plots from metabolic fingerprints reveal excellent separation between non-exposed and exposed cultures to toluene and DF1 loadings vector show that several regions derived from proteins and fatty acids contribute to this separation. An FT-IR approach would be a valuable tool as it can be employed to analyse cellular response rapidly (Correa et al. 2012), thereby allowing more cost effective and high-throughput experiments to be conducted. We have also performed GC–MS analysis to monitor metabolome changes in the cultures and the results revealed that the levels of several amino acids in the central metabolic pathways of *P. putida* DOT-T1E strains were increased in response to toluene stress. The production of ornithine in the presence of toluene could be considered as a major key element and linked directly to solvent tolerance mechanisms. Finally, the combination of metabolic fingerprinting and profiling with suitable multivariate analysis is a valuable method for investigating solvent adaptation mechanisms in these industrially and environmentally significant microorganisms.

## Electronic supplementary material

Below is the link to the electronic supplementary material.

Supplementary material 1 (DOCX 589 kb)
