# Supplementary material for: Metabolic analysis of the response of Pseudomonas putida DOT-T1E strains to toluene using Fourier transform infrared spectroscopy and gas chromatography mass spectrometry
Source: Metabolomics. 2016 Jun 21;12:112. doi: 10.1007/s11306-016-1054-1 (PMC4916193; doi:10.1007/s11306-016-1054-1)
Supplement: Supplementary file 1 — Supplementary material 1 (DOCX 589 kb) [file 11306_2016_1054_MOESM1_ESM.docx]

Metabolic analysis of the response of *Pseudomonas putida* DOT-T1E strains to toluene using Fourier transform infrared spectroscopy and gas chromatography mass spectrometry

Ali Sayqal, Yun Xu, Drupad K. Trivedi, Najla AlMasoud, David I. Ellis, Howbeer Muhamadali, Nicholas J. W. Rattray, Carole Webb and Royston Goodacre

**Supplementary Information: Experimental**

### Sampling and analysis of cell extracts by HPLC-UV

To investigate the role of efflux pumps which extrude toluene from *P. putida* cells, all bacterial cells were normalised to an optical density at 660 nm (OD_660_) of 0.2 in 50 mL of LB medium and then incubated in an orbital shaker for 4 h at 30^o^C and 200 rpm. Once *P. putida* cultures reached the mid-log phase, samples were divided into two groups. One group was challenged with 0.2% (*v/v*) toluene and the second group was kept as an unexposed control. All flasks were sealed with Suba-Seal and incubated for an additional 30 min.

Cells (45 mL) were pelleted by centrifugation (3000 ×*g*, 10 min, 1^o^C) and the supernatant was removed, while the cell pellets were washed once with 10 mL of 0.9% saline solution and centrifuged again to ensure the complete removal of LB medium. The pellets were suspended in 1.5 mL of 100% methanol and transferred into a fresh 2 mL Eppendorf tube. To permeabilize the cells, the freeze-thaw cycles liquid nitrogen method was performed three times according to the method of Winder *et al*. ([Winder *et al.* 2008](#_ENREF_2)). The samples were then pelleted by centrifugation (13500 ×*g*, 5 min) and an aliquot (1200 µL) of supernatant (intracellular extracts) was normalised according to OD_660_. Finally, an aliquot (300 µL) of intracellular extracts was placed in a LC vial and analysed by high-performance liquid chromatography (HPLC-UV).

All measurements were carried out using HPLC system (Agilent Technologies) equipped with an Agilent 1260 Infinity Quaternary Pump, auto-sampler and programmable UV Diode Array Detector. The output signal was monitored at 218 nm. The chromatographic separation was performed in a C18 column (100 x 4.6 mm) and the column temperature was maintained at 20 ^o^C. HPLC separations were carried out by injecting 15 μL with an isocratic mobile phase methanol (100%) at a flow rate of 1 mL min^-1^. The total analysis time was 30 min.

Supplementary Information: Results

Fig. S1 Chromatograms for (A) 0.01% toluene, (B) *P. putida* DOT-T1E (no toluene), exposed cells to 0.2% toluene (C) *P. putida* DOT-T1E, (D) *P. putida* DOT-T1E-PS28 and (E) *P. putida* DOT-T1E-18 obtained from HPLC-UV. Toluene eluted with retention time of 1.458 ± 0.003 min.

Fig. S2 Calibration curve obtained from toluene ranging from 0.001 to 1 mM for 3 replicates using HPLC-UV. Points are means of the 3 replicates and error bars are standard deviations.

Fig. S3 Box-whisker plot representing the toluene level in *P. putida* strains exposed to 0.2% (*v*/*v*) toluene for 4 replicates. The red lines indicate the median of the peak area. DOT-T1E is the wild type, DOT-T1E-PS28 is the mutant (lacking the TtgGHI pump) and DOT-T1E-18 is the mutant (lacking the TtgABC pump). Error bars are standard deviations of 4 replicates.

Fig.S4 Validated PC-DFA models of (A) *P. putida* DOT-T1E, (B) DOT-T1E-PS28, (C) DOT-T1E-18 upon toluene stress. Symbol coding: control with no toluene (circles), cells exposed to 0.1% (*v*/*v*) toluene (squares), toluene via gas phase (triangles), and toluene via gas phase and 0.1% (*v*/*v*) toluene (upside down triangles). Closed symbols represent the training set while open symbols represent the test set that was projected into the PC-DFA scores space constructed from the training set.

Fig. S5 Effect of oxidative stress on *P. putida* strains growth. Symbols and colours represent different strains. (Closed black diamonds) represents the wild-type DOT-T1E, (Closed red circle) the mutant DOT-T1E-PS28, and (Closed green triangles) the mutant DOT-T1E-18. (Solid lines) represent the growth curves of the control cells, while (dotted lines) cells exposed to oxidative stress.

Fig. S6 Schematic metabolic diagram of central carbon metabolism in *P. putida* DOT-T1E-PS28 adapted to toluene. Metabolites were detected and identified by GC-MS. Metabolites indicated in black were observed, while metabolites indicated in grey were not detected. (A) Represent the level of metabolites at 10 min after toluene exposure, and (B) at 60 min. Box-whisker plot showing the changes in metabolite levels in control and cells exposed to toluene for 4 *biological* replicates. The red lines indicate the median *m*/*z* intensity. Codes: control - no toluene (C), cells exposed to 0.1% (*v*/*v*) toluene (T), toluene gas (G), and toluene gas and 0.1% (*v*/*v*) toluene (GT).

Fig. S7 Schematic metabolic diagram of central carbon metabolism in *P. putida* DOT-T1E-18 adapted to toluene. Metabolites were detected and identified by GC-MS. Metabolites indicated in black were observed, while metabolites indicated in grey were not detected. (A) Represent the level of metabolites at 10 min after toluene exposure, and (B) at 60 min. Box-whisker plot showing the changes in metabolite levels in control and cells exposed to toluene for 4 *biological* replicates. The red lines indicate the median *m*/*z* intensity. Codes: control - no toluene (C), cells exposed to 0.1% (*v*/*v*) toluene (T), toluene gas (G), and toluene gas and 0.1% (*v*/*v*) toluene (GT).

**Table S1** Results from the toluene MIC experiments using *P. putida* DOT-T1E, DOT-T1E-PS28 and DOT-T1E-18. Culture growth was recorded after overnight incubation.

| *P. putida* strains | Toluene concentration (% (*v*/*v*)) | Toluene concentration (mM) | Growth (+/-)* |
| --- | --- | --- | --- |
| DOT-T1E | 0 | 0 | + |
|  | 0.3 | 30 | + |
|  | 0.5 | 50 | + |
|  | 0.7 | 70 | + |
|  | 0.8 | 80 | + |
|  | 1 | 100 | + |
|  | 2 | 200 | + |
|  | 3 | 300 | ± or + |
|  | 4 | 400 | ± |
|  | 5 | 500 | - |
| DOT-T1E-PS28 | 0 | 0 | + |
|  | 0.3 | 30 | + |
|  | 0.5 | 50 | ± or + |
|  | 0.7 | 70 | ± |
|  | 0.8 | 80 | - |
|  | 1 | 100 | - |
|  | 2 | 200 | - |
|  | 3 | 300 | - |
|  | 4 | 400 | - |
|  | 5 | 500 | - |
| DOT-T1E-18 | 0 | 0 | + |
|  | 0.3 | 30 | ± or + |
|  | 0.5 | 50 | ± |
|  | 0.7 | 70 | - |
|  | 0.8 | 80 | - |
|  | 1 | 100 | - |
|  | 2 | 200 | - |
|  | 3 | 300 | - |
|  | 4 | 400 | - |
|  | 5 | 500 | - |

*(+) indicates growth, (±) slight growth, and (-) no growth

**Table S2** The level of toluene in *P. putida* strains

| *P. putida* strains | Toluene concentration (µM) |
| --- | --- |
| DOT-T1E | 33 ± 2 |
| DOT-T1E-PS28 | 71 ± 11 |
| DOT-T1E-18 | 277 ± 18 |

**Table S3** List of the top 30 significant variables from MB-PCA loading.

| Conditions | time | strains |
| --- | --- | --- |
| 7 | 13 | 4 |
| 12 | 15 | 14 |
| 13 | 16 | 15 |
| 14 | 19 | 19 |
| 15 | 22 | 20 |
| 18 | 23 | 22 |
| 19 | 26 | 23 |
| 20 | 31 | 31 |
| 22 | 33 | 33 |
| 23 | 38 | 36 |
| 25 | 44 | 43 |
| 26 | 45 | 44 |
| 28 | 49 | 45 |
| 30 | 50 | 49 |
| 31 | 52 | 52 |
| 35 | 56 | 62 |
| 36 | 57 | 63 |
| 43 | 59 | 64 |
| 44 | 60 | 69 |
| 45 | 61 | 75 |
| 49 | 63 | 80 |
| 52 | 65 | 88 |
| 53 | 68 | 90 |
| 54 | 69 | 97 |
| 56 | 74 | 99 |
| 57 | 75 | 103 |
| 58 | 76 | 105 |
| 59 | 82 | 107 |
| 60 | 83 | 110 |
| 61 | 84 | 111 |

**Table S4** List of the top 30 significant variables from *N*-way ANOVA test.

| Order of most significant | Conditions | | | time | | | strains | | |
| --- | --- | --- | --- | --- | --- | --- | --- | --- | --- |
|  | variable | *p*-value | FDR | variable | *p*-value | FDR | variable | *p*-value | FDR |
| 1 | 25 | 8.78E-16 | 8.96E-15 | 13 | 9.08E-05 | 2.59E-03 | 14 | 2.15E-25 | 2.15E-25 |
| 2 | 29 | 7.10E-10 | 3.62E-09 | 99 | 4.81E-05 | 2.74E-03 | 99 | 6.98E-24 | 6.98E-24 |
| 3 | 101 | 6.09E-08 | 1.24E-07 | 91 | 3.16E-04 | 5.99E-03 | 36 | 1.92E-19 | 1.92E-19 |
| 4 | 109 | 3.94E-08 | 1.34E-07 | 58 | 10.85E-04 | 1.55E-02 | 20 | 1.85E-17 | 1.85E-17 |
| 5 | 45 | 5.58E-08 | 1.42E-07 |  |  |  | 64 | 1.38E-12 | 1.38E-12 |
| 6 | 91 | 2.39E-07 | 4.06E-07 |  |  |  | 62 | 1.27E-11 | 1.27E-11 |
| 7 | 36 | 6.34E-07 | 9.24E-07 |  |  |  | 25 | 1.91E-11 | 1.91E-11 |
| 8 | 43 | 1.67E-06 | 1.70E-06 |  |  |  | 13 | 2.24E-10 | 2.24E-10 |
| 9 | 99 | 1.59E-06 | 1.80E-06 |  |  |  | 111 | 1.94E-09 | 1.94E-09 |
| 10 | 108 | 1.48E-06 | 1.89E-06 |  |  |  | 15 | 2.17E-09 | 2.17E-09 |
| 11 | 62 | 2.86E-06 | 2.65E-06 |  |  |  | 4 | 1.16E-08 | 1.16E-08 |
| 12 | 14 | 3.17E-06 | 2.70E-06 |  |  |  | 16 | 1.38E-08 | 1.38E-08 |
| 13 | 7 | 5.95E-06 | 4.67E-06 |  |  |  | 45 | 1.53E-08 | 1.53E-08 |
| 14 | 30 | 8.96E-06 | 6.53E-06 |  |  |  | 91 | 3.96E-08 | 3.96E-08 |
| 15 | 54 | 1.24E-05 | 8.43E-06 |  |  |  | 43 | 1.21E-07 | 1.21E-07 |
| 16 | 111 | 1.43E-05 | 9.09E-06 |  |  |  | 72 | 2.14E-07 | 2.14E-07 |
| 17 | 34 | 2.25E-05 | 1.35E-05 |  |  |  | 97 | 5.28E-07 | 5.28E-07 |
| 18 | 114 | 1.85E-04 | 9.95E-05 |  |  |  | 21 | 1.09E-06 | 1.09E-06 |
| 19 | 112 | 1.77E-04 | 1.01E-04 |  |  |  | 81 | 1.83E-06 | 1.83E-06 |
| 20 | 52 | 2.43E-04 | 1.12E-04 |  |  |  | 73 | 5.31E-06 | 5.31E-06 |
| 21 | 28 | 2.42E-04 | 1.17E-04 |  |  |  | 108 | 5.83E-06 | 5.83E-06 |
| 22 | 87 | 2.66E-04 | 1.18E-04 |  |  |  | 65 | 6.84E-06 | 6.84E-06 |
| 23 | 44 | 2.33E-04 | 1.19E-04 |  |  |  | 87 | 1.25E-05 | 1.25E-05 |
| 24 | 58 | 3.13E-04 | 1.33E-04 |  |  |  | 47 | 1.38E-05 | 1.38E-05 |
| 25 | 41 | 3.37E-04 | 1.38E-04 |  |  |  | 110 | 1.45E-05 | 1.45E-05 |
| 26 | 9 | 3.75E-04 | 1.47E-04 |  |  |  | 48 | 1.45E-05 | 1.45E-05 |
| Order of most significant | Conditions | | | time | | | strains | | |
|  | variable | *p*-value | FDR | variable | *p*-value | FDR | variable | *p*-value | FDR |
| 27 | 64 | 5.68E-04 | 2.07E-04 |  |  |  | 92 | 1.81E-05 | 1.81E-05 |
| 28 | 72 | 5.5E-04 | 2.08E-04 |  |  |  | 88 | 3.26E-05 | 3.26E-05 |
| 29 | 12 | 10.13E-04 | 3.56E-04 |  |  |  | 80 | 3.43E-05 | 3.43E-05 |
| 30 | 96 | 11.04E-04 | 3.75E-04 |  |  |  | 113 | 3.54E-05 | 3.54E-05 |

**Table S5** A list of detected metabolites by GC-MS. All identifications are based on minimum metabolite reporting standards ([Sumner *et al.* 2007](#_ENREF_1)).

| Variables ID | RT | RI | Metabolite | MSI ID level |
| --- | --- | --- | --- | --- |
| 7 | 389.978 | 1100 | Alanine | 1 |
| 12 | 470.578 | 1227.9 | Valine | 1 |
| 15 | 479.478 | 1242 | Leucine | 1 |
| 18 | 498.828 | 1272.7 | Isoleucine | 1 |
| 24 | 529.928 | 1322.1 | Glycine | 1 |
| 26 | 545.628 | 1347 | Phosphate | 1 |
| 28 | 555.528 | 1362.7 | Threonine | 1 |
| 30 | 563.478 | 1375.4 | Serine | 2 |
| 36 | 617.228 | 1460.7 | Uracil | 1 |
| 41 | 658.228 | 1535.7 | Aspartic acid | 1 |
| 44 | 692.778 | 1611.6 | Methionine | 1 |
| 45 | 702.478 | 1632.9 | Arabinose | 1 |
| 47 | 717.278 | 1665.4 | Glutamine | 1 |
| 49 | 732.628 | 1699.2 | Pyroglutamic acid | 1 |
| 52 | 749.528 | 1736.3 | Phenylalanine | 2 |
| 54 | 768.978 | 1779.1 | Ornithine | 1 |
| 56 | 775.128 | 1792.6 | Mannose | 2 |
| 65 | 796.628 | 1839.8 | Citric Acid | 2 |
| 68 | 812.028 | 1873.7 | Lysine | 1 |
| 82 | 864.578 | 2007.7 | Tyrosine | 2 |
| 89 | 886.878 | 2066.9 | Tyramine | 1 |
| 92 | 907.178 | 2120.8 | Adenine | 1 |
| 100 | 966.728 | 2278.9 | Octadecanoic acid | 1 |
| 112 | 1046.68 | 2491.2 | Tryptophan | 2 |

Code: ID, identifier on plots; RT, retention time; RI, retention index; MSI, Metabolomics Standards Initiative

**References**

Sumner, L. W., Amberg, A., Barrett, D., Beale, M. H., Beger, R., Daykin, C. A., Fan, T. W. M*., et al.* (2007). Proposed minimum reporting standards for chemical analysis. *Metabolomics*, 3, 211-221.

Winder, C. L., Dunn, W. B., Schuler, S., Broadhurst, D., Jarvis, R., Stephens, G. M., & Goodacre, R. (2008). Global metabolic profiling of *Escherichia coli* cultures: an evaluation of methods for quenching and extraction of intracellular metabolites. *Analytical Chemistry*, 80, 2939-2948.
